# Detecting white spot lesions on post-orthodontic oral photographs using deep learning based on the YOLOv5x algorithm: a pilot study

**DOI:** 10.1186/s12903-024-04262-1

**Published:** 2024-04-24

**Authors:** Pelin Senem Ozsunkar, Duygu Çelİk Özen, Ahmed Z Abdelkarim, Sacide Duman, Mehmet Uğurlu, Mehmet Rıdvan Demİr, Batuhan Kuleli, Özer Çelİk, Busra Seda Imamoglu, Ibrahim Sevki Bayrakdar, Suayip Burak Duman

**Affiliations:** 1 Department of Paediatric Dentistry, Faculty of Dentistry, Inonu University, Malatya, Turkey; 2 Department of Oral and Maxillofacial Radiology, Faculty of Dentistry, Inonu University, Malatya, Turkey; 3 Division of Oral & Maxillofacial Radiology, College of Dentistry, The Ohio State Universiy, Columbus, OH USA; 4 Department of Orthodontics, Faculty of Dentistry, Eskisehir Osmangazi University, Eskisehir, Turkey; 5 Department of Orthodontics, Faculty of Dentistry, Ataturk University, Erzurum, Turkey; 6 Department of Mathematics-Computer, Eskişehir Osmangazi University Faculty of Science, Eskişehir, Turkey; 7 Department of Orthodontics, Hamidiye Faculty of Dentistry, University of Health Sciences, Istanbul, Turkey; 8 Department of Oral and Maxillofacial Radiology, Faculty of Dentistry, Eskisehir Osmangazi University, Eskisehir, Turkey; 9 Department of Oral and Maxillofacial Radiology, Faculty of Dentistry, Inonu University, Malatya, 44280 Turkey

**Keywords:** Artificial intelligence, Deep learning, Dentistry, Photography, White spot lesions

## Abstract

**Background:**

Deep learning model trained on a large image dataset, can be used to detect and discriminate targets with similar but not identical appearances. The aim of this study is to evaluate the post-training performance of the CNN-based YOLOv5x algorithm in the detection of white spot lesions in post-orthodontic oral photographs using the limited data available and to make a preliminary study for fully automated models that can be clinically integrated in the future.

**Methods:**

A total of 435 images in JPG format were uploaded into the CranioCatch labeling software and labeled white spot lesions. The labeled images were resized to 640 × 320 while maintaining their aspect ratio before model training. The labeled images were randomly divided into three groups (Training:349 images (1589 labels), Validation:43 images (181 labels), Test:43 images (215 labels)). YOLOv5x algorithm was used to perform deep learning. The segmentation performance of the tested model was visualized and analyzed using ROC analysis and a confusion matrix. True Positive (TP), False Positive (FP), and False Negative (FN) values were determined.

**Results:**

Among the test group images, there were 133 TPs, 36 FPs, and 82 FNs. The model’s performance metrics include precision, recall, and F1 score values of detecting white spot lesions were 0.786, 0.618, and 0.692. The AUC value obtained from the ROC analysis was 0.712. The mAP value obtained from the Precision-Recall curve graph was 0.425.

**Conclusions:**

The model’s accuracy and sensitivity in detecting white spot lesions remained lower than expected for practical application, but is a promising and acceptable detection rate compared to previous study. The current study provides a preliminary insight to further improved by increasing the dataset for training, and applying modifications to the deep learning algorithm.

**Clinical revelance:**

Deep learning systems can help clinicians to distinguish white spot lesions that may be missed during visual inspection.

**Supplementary Information:**

The online version contains supplementary material available at 10.1186/s12903-024-04262-1.

## Background

White spot lesions, which are opaque and present in the early stages of caries, indicate that the enamel decalcification. Patients who use dental appliances, receive orthodontic treatment or are at high risk of developing caries have a higher incidence of white spot lesions [1]. The lesions are primarily seen in the cervical and middle third of the teeth labial surface. The most affected teeth are maxillary and mandibular first molars, maxillary lateral, mandibular lateral, and mandibular canines [2]. White spot lesions are a significant obstacle for clinicians, as they impact the appearance and signify the beginning stages of caries formation due to subsurface demineralization. These lesions have an opaque, chalky appearance located in areas where orthodontic brackets were once present [1, 2].

Various approaches can be used to diagnose white spot lesions, ranging from traditional methods (visual inspection, probe examination, etc.) to advanced technologies (Digital Laser Fluorescence, Fiber Optic Transillumination (FOTI), etc.). Pitts [3] stated that the instruments and methods used to diagnose lesions should be reliable, easy to apply, non-invasive, and able to measure the size and activity of the lesion. Archival dental photographs can be an effective tool in the diagnosis and treatment decision of post-orthodontic white spot lesions. Creating independent diagnostic methods can become feasible with intelligent image analysis methods. Artificial intelligence (AI) can automate the identification and assessment of diagnostic data in medicine and dentistry, allowing for the development of independent diagnostic procedures [4, 5].

The subfield of artificial intelligence known as machine learning has demonstrated its efficacy in computer-aided diagnostic support tasks. In this field, algorithms learn patterns and structures in data through training and can then be applied to make predictions on unseen data during inference. Deep learning, which involves multilayer neural networks, is a popular area in machine learning. In particular, Convolutional Neural Networks (CNNs) are commonly used for complex data structures, such as images, to learn non-linear patterns. Deep CNNs, if trained on a large enough image dataset, can be used to detect and discriminate targets with similar but not identical appearances. To develop a deep CNN for detecting white spot lesions in clinical photographs and to determine whether the model could also distinguish between different white spots, Askar et al. [6] used a particular type of CNN titled SqueezeNet. They discovered that it accurately detected white spot lesions, especially fluorosis. However, in the same study, to overcome the limited sample size in non-fluorotic lesions, lesions with remarkably different appearances (caries, hypomineralized lesions) were grouped. They emphasized that detecting a severe fluorotic lesion may be more difficult than detecting a mild lesion, and therefore more detailed specific classifications are needed [6].

You Only Look Once (YOLO) was a CNN-based object detection algorithm that reframes directly from image pixels to bounding box coordinates and class probabilities as a single regression problem. The YOLO design allows for end-to-end training and real-time speeds while maintaining high average accuracy [7]. There are different versions of this algorithm used in dentistry in areas such as early detection of oral cancers [8], identification and detection of madibular fractures [9], diagnosis and classification of temporomandibular joint osteoarthritis [10], and early caries detection [11, 12]. The aim of this study is to evaluate the post-training performance of the CNN-based YOLOv5x algorithm in the detection of white spot lesions in post-orthodontic oral photographs with the limited number of data we have and to make a preliminary study for fully automated models that can be clinically integrated in the future.

## Methods

### Ethical considerations

The study was approved by the Eskisehir Osmangazi University Faculty of Dentistry Ethics Committee (ethics number 020–798), All procedures involving human participants were conducted following the Declaration of Helsinki.

### Sample size calculation

On the basis of the power analysis that was carried out, it was concluded that 435 images would be sufficient to obtain reliable results using a paired two-sample t-test with a power of 95%, a margin of error of 5% and an effect size of dz = 0.17 [13, 14].

### Study design

This diagnostic study used intraoral clinical photographs taken for orthodontic treatment to detect white spot lesions in patients aged 16 to 62. The Standards for Reporting of Diagnostic Accuracy Studies (STARD) [15] guidelines and the Checklist for Artificial Intelligence in Medical Imaging (CLAIM) [16] were followed in reporting this study.

### Intraoral photographs

Photographs were taken after debonding and cleaning the teeth of plaque, blood, saliva, or filling material. Bilateral retractors were used during the photography. Shots were taken with a digital camera (Canon EOS 70D, DSLM, Tokyo, Japan) using standard camera settings (shutter speed 1/80 and aperture settings f44) under appropriate lighting conditions, resolution, and exposure settings. The patient bit in centric occlusion for the frontal image. The frame was adjusted to show the maximum number of teeth, including the second molars, without showing cheek retractors or cotton pads. The focus was adjusted to the lateral teeth in anterior shots and to the middle of the canines and premolars in lateral shots to attain the correct depth of field. Inadequate images that could affect image quality, such as out-of-focus images, underexposed, or overexposed images, were excluded. Clinical photographs with additional cavitated caries and hypoplasic teeth due to developmental disorders (amelogenesis imperfecta, dentinogenesis imperfecta, MIH, etc.) were also excluded from the study (determined by comparison with the initial photographs).

### Classification of teeth with white spot lesions

Dentists were trained on 50 different photographs to distinguish white spot lesions from developmental hypoplasia for calibration purposes and discussed until consensus was reached. A total of 435 high-quality clinical photographs of anterior and posterior permanent teeth in JPG format, ranging in size from 1 to 33 Mb, that were agreed to contain white spot lesions were collected and uploaded to the CranioCatch labelling software program. The dentists who labelling were personally trained and calibrated on how to use the tool and how to annotate white spot lesions as well as how to distinguish them from each other prior to the labelling tasks. Each white spot lesion in the photos included by the two dentists (P.S.O - pediatric dentistry, M.U - orthodontist) was labeled polygonally, following its outer boundaries. White spot lesion areas that appeared on more than one tooth in a photograph or on different surfaces of a tooth were labeled separately, resulting in a total of 1985 labels.

The labeled images were then independently checked by two dentists (S.D-pediatric dentist, S.B.D-dentomaxillofacial radiologist) with over ten years of clinical practice and scientific experience. Each intraoral image was re-evaluated and, in case of discrepant findings, discussed until a consensus was reached. Since the images were not independently seen twice by the same expert and the examiner (in the second round) was always aware of the labeling of the first expert, intra-oral or inter-oral reliability could not be calculated.

### The model

YOLO [7] approaches object detection as a single regression issue, bypassing the pipeline of region proposal, classification, and duplication elimination, in contrast to other object detection algorithms that have been previously established. Images are reduced in size and resolution using YOLO techniques, which then run a single CNN on the images and output the detection results based on the model’s confidence threshold. To increase the variety of the input photographs and increase the model’s robustness for object detection in various contexts, online data augmentation is incorporated in the YOLO system. In this study, deep learning was performed using the YOLOv5x architecture pre-trained on the original COCO 2017 dataset, powered by the PyTorch library and Python open-source programming language (v.3.6.1; Python Software Foundation, Wilmington, DE, USA). The Dental-AI Laboratory of Eskisehir Osmangazi University Faculty of Dentistry used Dell PowerEdge T640 Calculation Server (Dell Inc., Austin, TX, USA), Dell PowerEdge T640 GPU Calculation Server (Dell Inc., TX, USA), and Dell PowerEdge R540 Storage Server (Dell Inc., TX, USA) for the training process.

### Training of the deep learning based CNN (test method)

In deep learning, ground truth refers to the precise reference data used for training, evaluating or measuring the accuracy of a model. This data is usually that has been manually labeled or considered correct by humans. The ground truth represents the targeted outcomes in the model’s learning process and is used to support the model’s training, evaluate its performance and report results. In this study, Non-maximum Suppression (NMS) method was used for cases where more than one independent segmentation was performed. Non-maximum suppression (NMS) is a post-processing technique commonly used in object detection algorithms. In cases with multiple detection results (such as bounding box or segmentation results), it is used to filter these results and select the best results. In this algorithm, the confidence value of each detection result is first checked. Select the detection with the highest probability and save the bounding box or segmentation result for that detection. The remaining detection results are compared with the results that largely coincide with the highest probability selected in the previous step. Results above a certain threshold of overlap (e.g. 50%) are eliminated. That is, only the best result is selected from the probabilistic results for the same object. This process is repeated for all detection results.

**Fig. 1:**
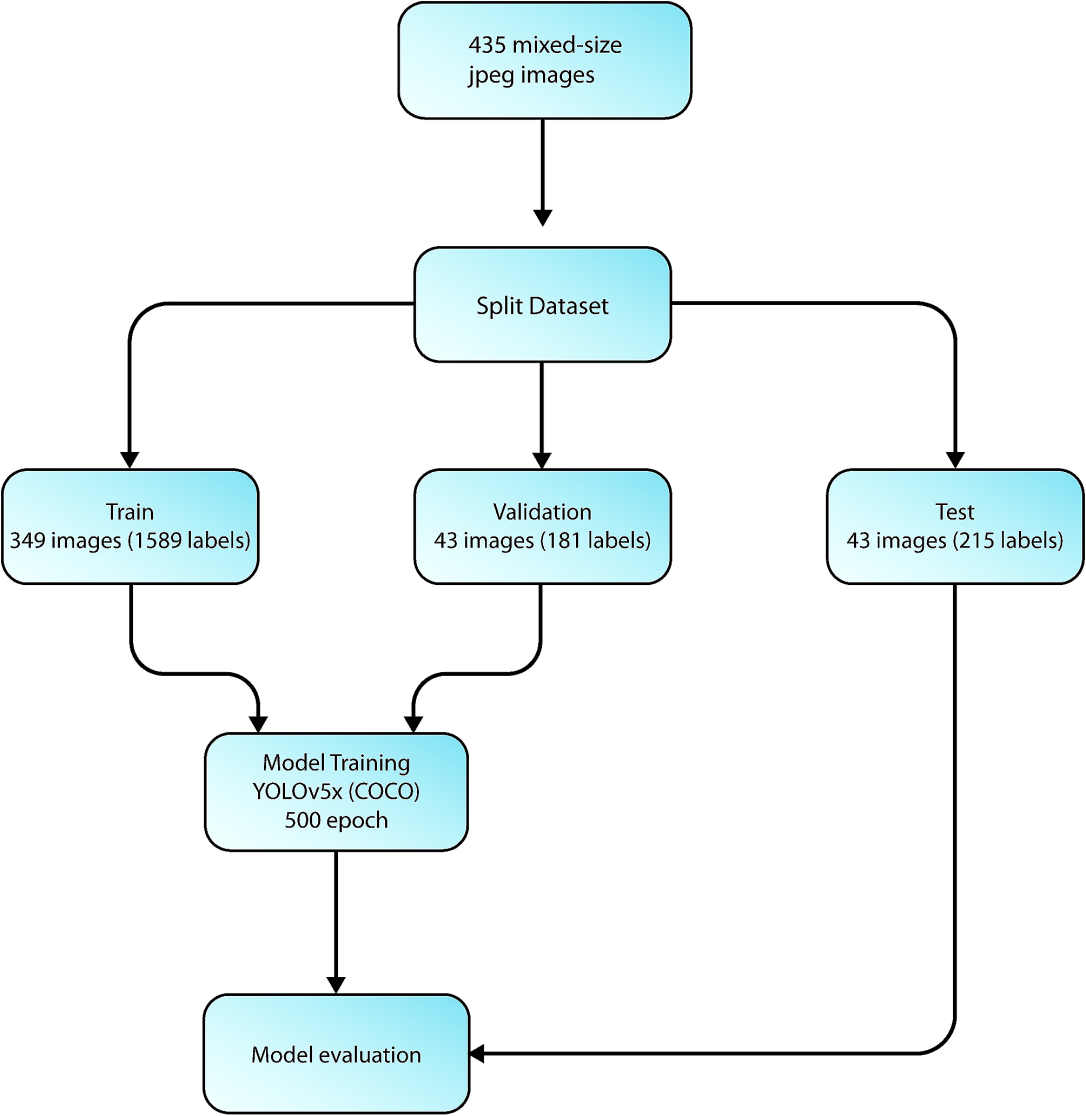
Diagram of the white spot lesions segmentation model development steps

The labeled images were resized to 640 × 320 while maintaining their aspect ratio before model training. Before training, the whole image set (1985 labels on 435 intraoral photos) was divided into train (1589 labels on 349 intraoral photos), validation (181 labels on 43 intraoral photos) and a test group (215 labels on 43 intraoral photos) (Fig. 1). Each label on an image represents a different lesion region. The CNN model had no information about other groups during training; its performance was evaluated only on an independent test set.

**Fig. 2:**
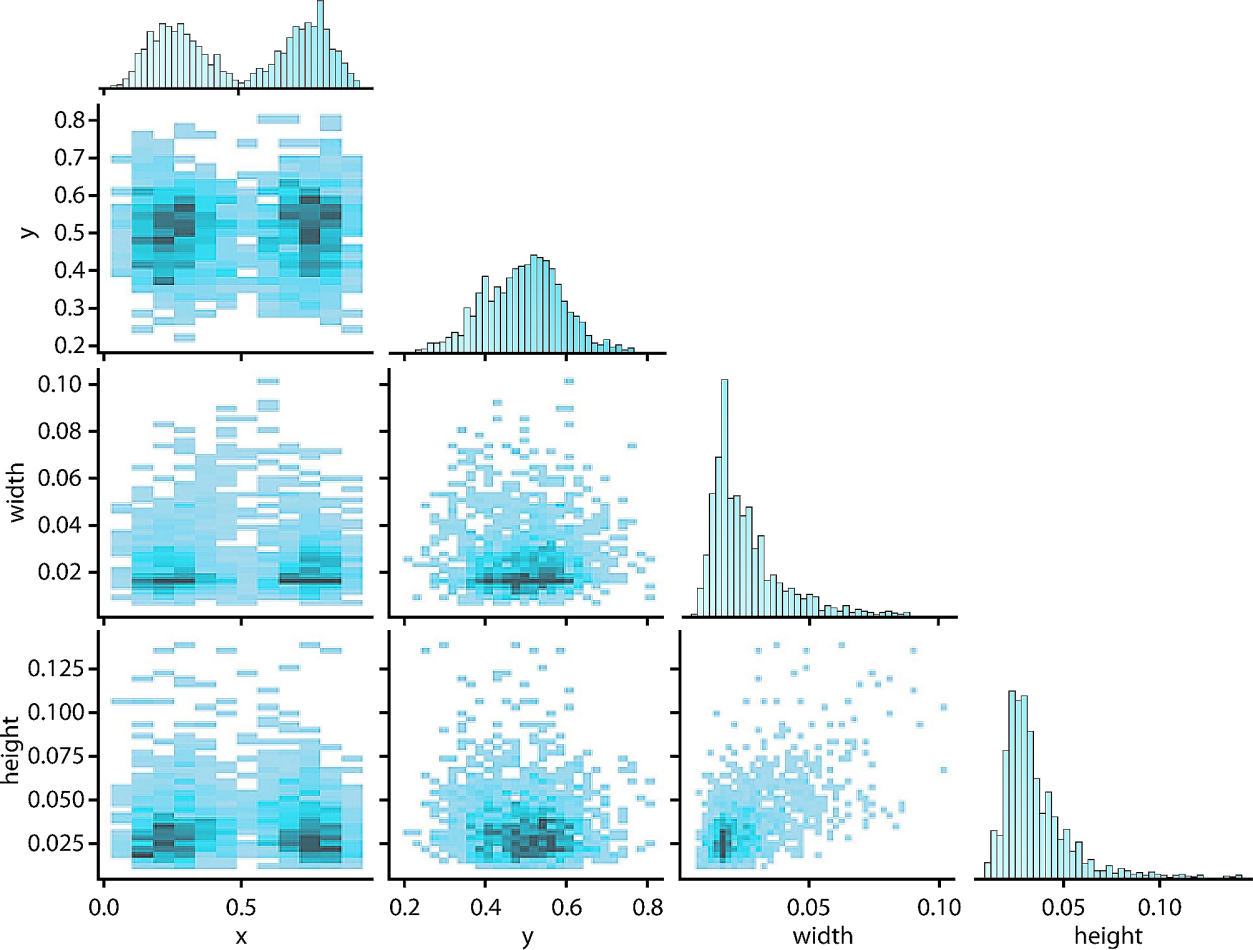
Correlogram showing YOLOv5x performance

The optimized deep learning model was evaluated on an independent test dataset after it had been trained on the images using 500 epochs and a learning rate of 0.01. The best model was then stored. The performance of YOLOv5x is shown in the correlogram in Fig. 2. The size of the points in the graph represents the confidence score of YOLOv5x for each object. The bigger the point, the higher the confidence score.

### Statistical analysis

The segmentation performance of the tested deep learning model was visualized and analyzed using Receiver Operating Characteristic (ROC) analysis and a confusion matrix Precision, recall, and F1 score metrics were calculated using these values.

#### TP

white spot lesion correctly detected and segmented.

#### FP

white spot lesion detected but incorrectly segmented.

#### FN

white spot lesion incorrectly detected and segmented.

$${\text{Precision:}}\;\frac{{TP}}{{TP + FP}}$$

$${\text{Recall:}}\,\frac{{TP}}{{TP + FN}}$$

$$F1 - Score:\frac{{2x\,PrecisionxRecall}}{{Precision + Recall}}$$

## Results

**Fig. 3:**
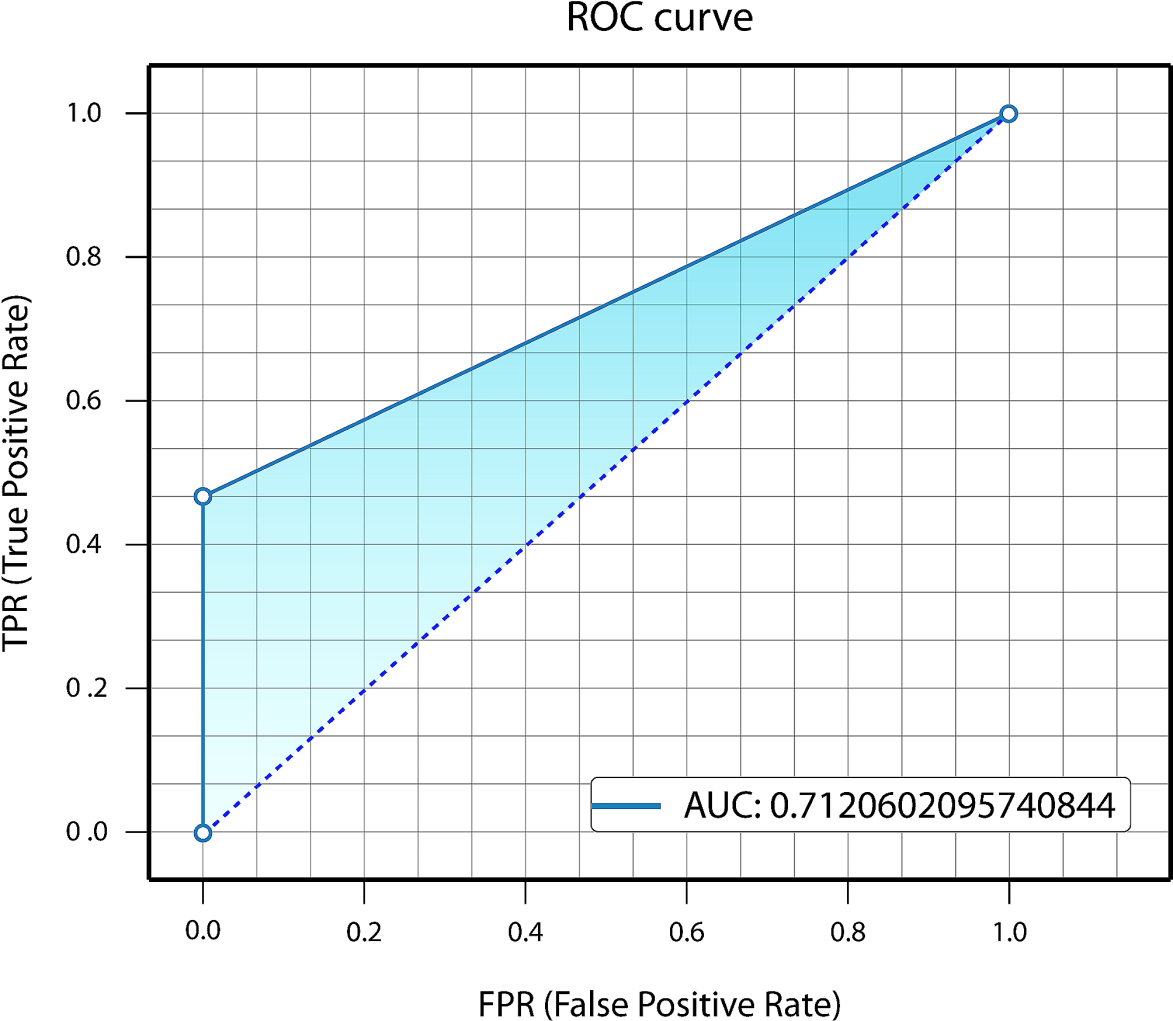
White spot lesions segmentation on intraoral photograph using the AI model. The graphs of the ROC analysis results for white spot lesions segmentation

**Fig. 4:**
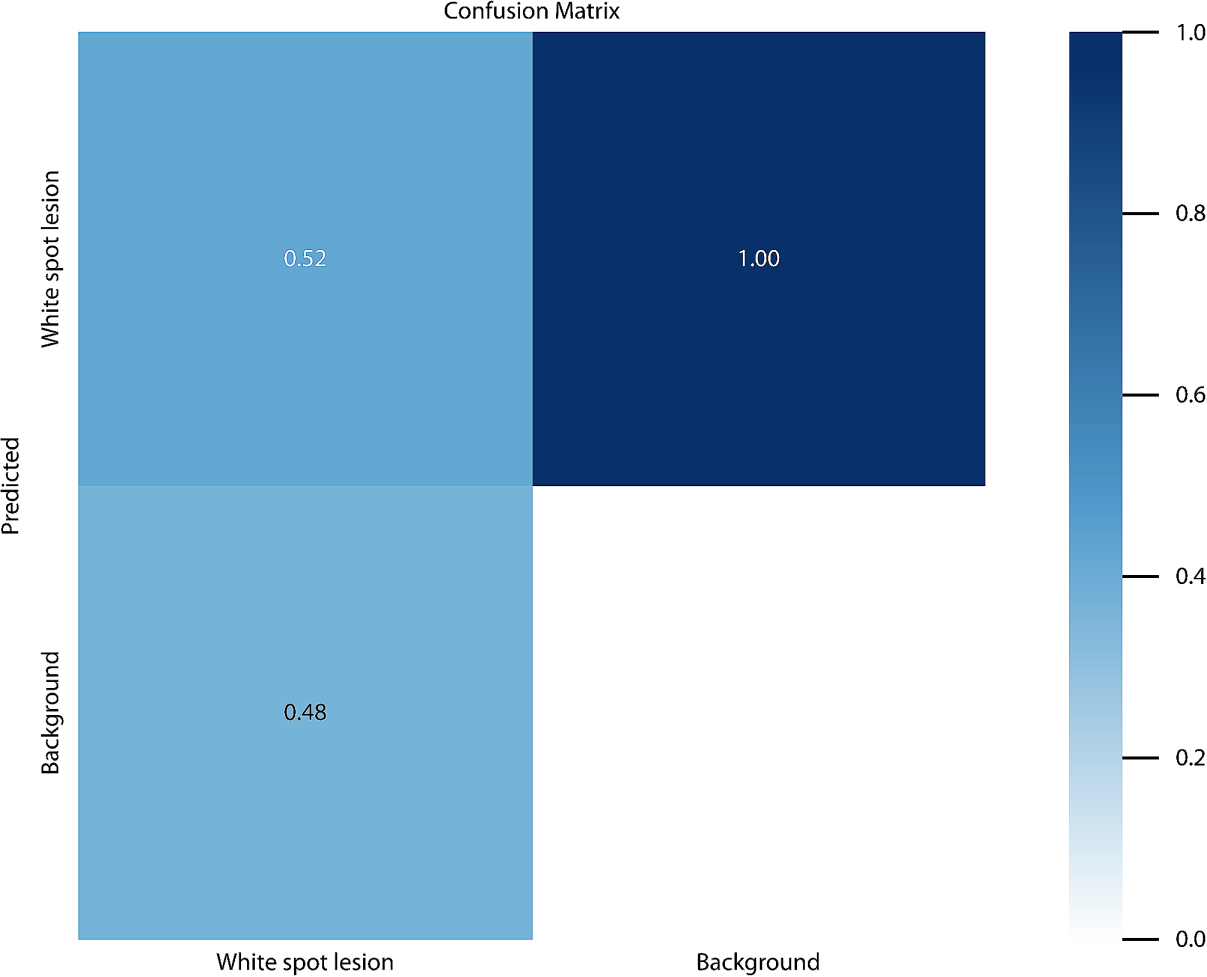
Confusion matrix showing the YOLOv5x segmentation performance for the test sample

**Fig. 5:**
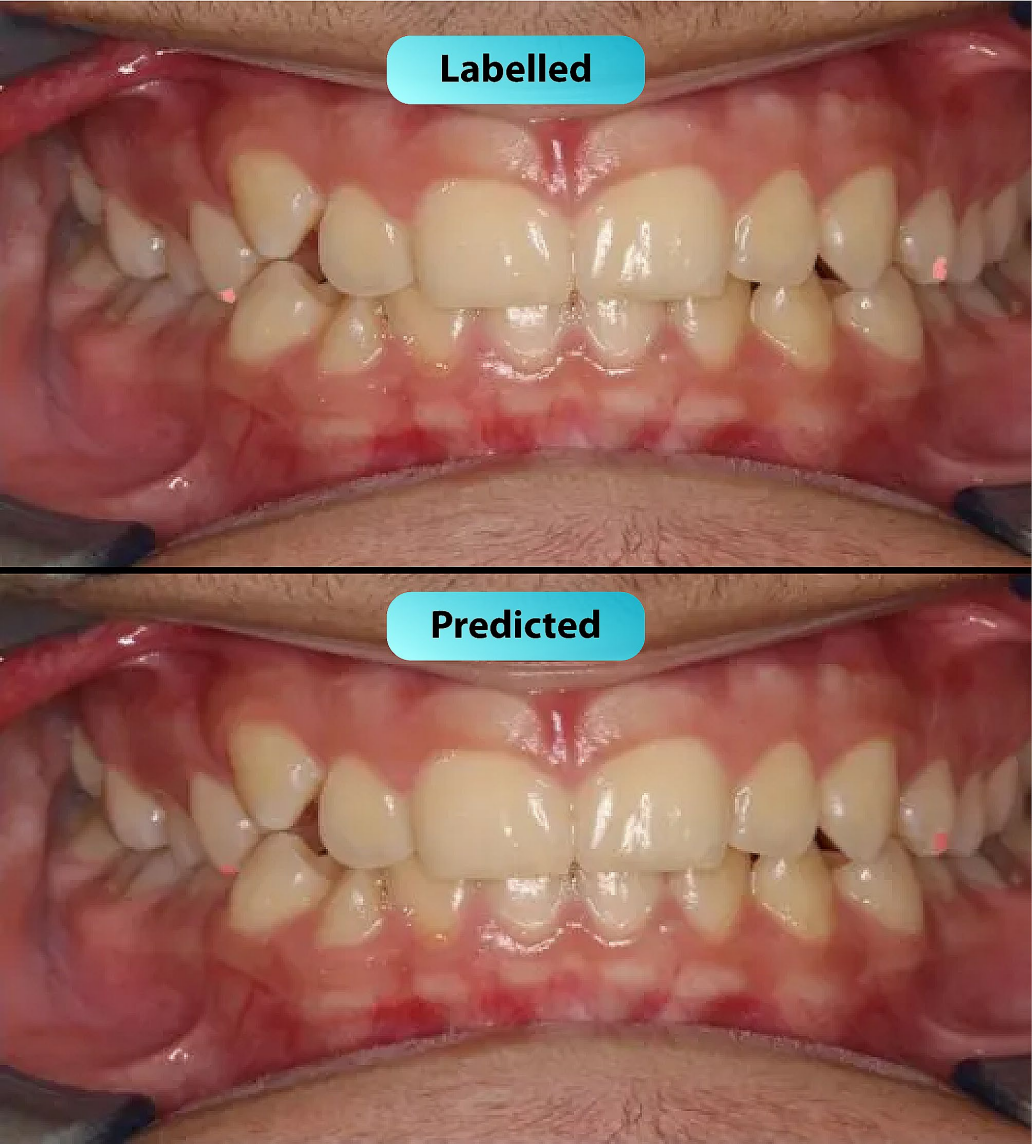
The graphs of the ROC analysis results for white spot lesions segmentation

**Fig. 6:**
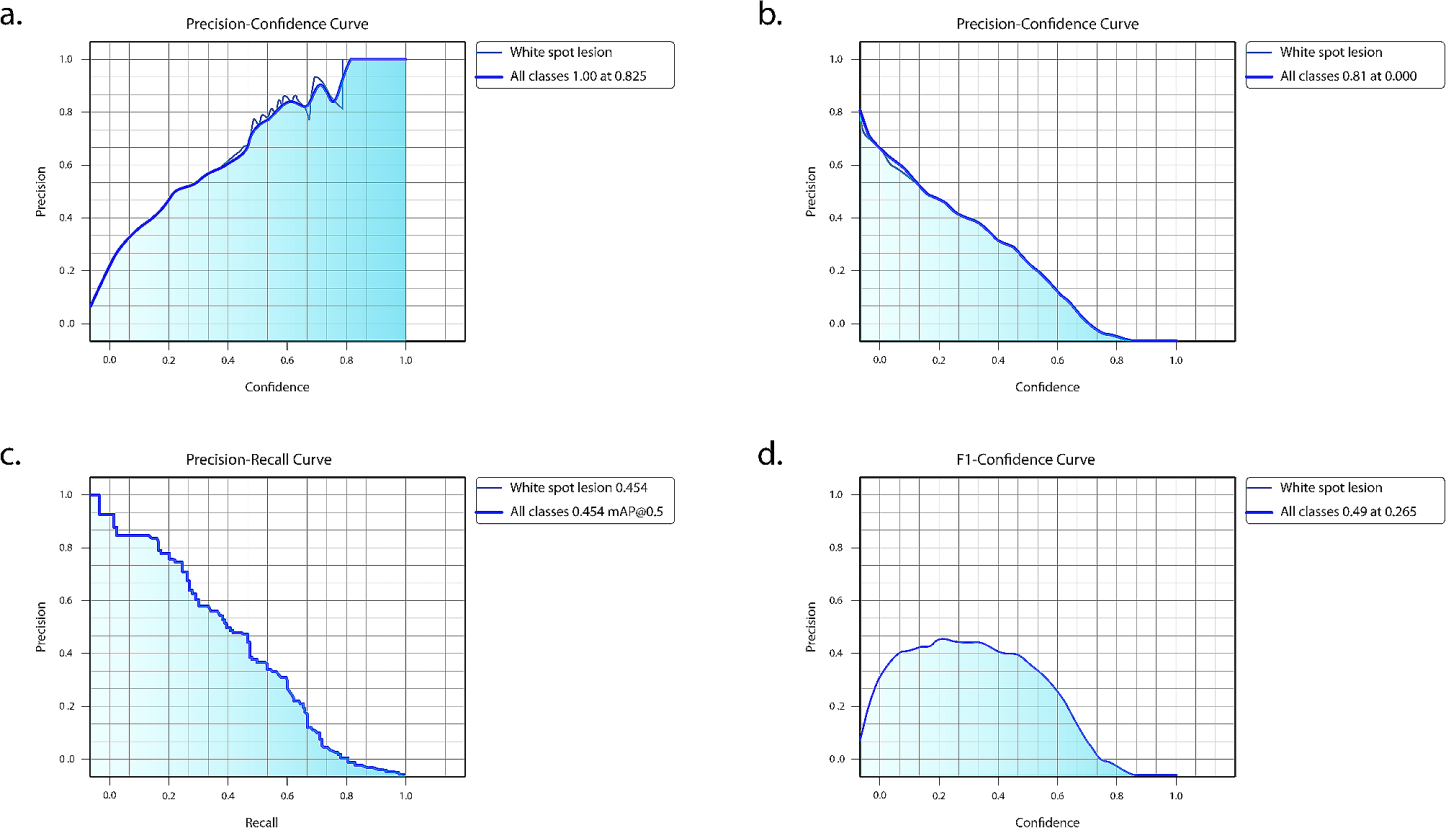
**a**. Precision-Confidence Curve. **b.** Recall-Confidence Curve. **c.** F1- Confidence Curve. **d**. Precision-Recall Curve

After training the deep learning-based model, white spot lesions were successfully segmented (Fig. 3). Confusion matrix metrics are shown in Fig. 4. The model’s performance metrics include a precision value of 0.786, a recall value of 0.618, and an F1 score of 0.692 (Table 1). The AUC value, calculated as the area under the ROC curve in the graph obtained from the ROC analysis, was 0.712 (Fig. 5). Figure 6 for the Precision-Confidence curve, Recall-Confidence curve, F1-Confidence curve, and Precision-Recall Curve graphs. The mAP value obtained from the Precision-Recall curve graph was 0.425.

**Table 1:** Predictive performance measurement using the AI model for white spot lesions segmentation in test data

Model	TP	FP	FN	Recall	Precision	F1 Score
White Spot Lesion Segmentation	133	36	82	0.618	0.786	0.692

## Discussion

Early caries symptoms include opaque white spot lesions, indicating decalcification of the enamel. Even for experienced dentists, identifying the early caries is challenging. The sensitivity and specificity of photographic visual inspection were calculated as 67% and 79%, respectively, in a systematic review and meta-analysis aimed at examining the efficacy of fluorescence-based methods, visual inspections, and photographic visual inspections in detecting initial caries [17]. Existing literature includes studies using intraoral photographs and YOLOv3 algorithms that show a promising detection rate [11, 12]. For instance, a study leveraged the YOLOv3 algorithm to accurately detect dental caries, focusing on primary caries, using oral photographs captured via mobile phones. The findings of the study revealed satisfactory results [11]. In a other study, a training dataset comprising 1902 dental photographs taken using smartphones was employed to diagnose the stages of smooth surface caries using four deep learning models, including Faster R-CNNs, YOLOv3, RetinaNet, and Single-Shot Multi-Box Detector (SSD). The study found that the YOLOv3 model outperformed the other three models, achieving a sensitivity rate of 87.4% for caries with cavities. The YOLOv3 algorithm also generated fewer candidate frames than Faster R-CNN, as it does not require a two-phase detection process [12]. However, the accuracy and precision of all four models in detecting initial caries were lower than expected for practical applications, with YOLOv3 and Faster R-CNN achieving detection achieving sensitivity rates of 36.9% and 26%, respectively [12]. Additionaly, The YOLO algorithm generates candidate frames much less than Faster-RCNN and does not require 2 stages to complete the detection task. Therefore, these characteristics make the YOLO algorithm faster, capable of achieving real-time response levels, and more suitable for the application scenario of AI-assisted diagnosis of dental caries [18]. In this study, in the detection of white spot lesions, a more satisfactory result was found for the YOLOV5x model, one of the higher versions of YOLOv3, with a sensitivity level of 61%. This is an encouraging development in that the upgraded Yolo algorithms potentially offer faster processing times, real-time response rates and greater suitability for AI-assisted diagnostic applications.

YOLOv5 is a high-performance and versatile target detection algorithm available in four versions: YOLOv5s, YOLOv5m, YOLOv5l, and YOLOv5x, which differ in network width and depth. Although these models share similar structures, they have distinguishing features such as different numbers of convolutional layers. Increasing convolutional layers allows for a thicker feature map and strengthens the network’s ability to learn to extract features [19]. YOLOv5 has a rapid detection model, with a runtime of only 0.07 s per frame [8]. This study selected the YOLOv5x model due to its more significant convolutional layers and feature map than other versions. Tanriver et al. [20], studies in which oral lesions at photographs were evaluated with different versions of YOLOv5 using U-Net architectures, YOLOv5l model, a single-stage object detector for detecting oral lesions, showed the best performance among all versions with an extraction speed of 10.6 ms per image on the Tesla T4 graphics processing unit. In the same study, the use of YOLOv5l to detect lesion sites from the whole image and EfficientNet-b4 to classify the detected lesion site into three categories has been suggested [20]. In another study aiming to evaluate the performance of deep CNN algorithms for classification and detection of oral potentially malignant disorders and oral squamous cell carcinomas in oral photographic images, the lowest performance model in detection of oral potentially malignant disorders is YOLOv5 which achieved a precision of 0.74, a recall of 0.39, a F1 score of 0.51 and an AUC of 0.34. However, these rates in the detection of oral squamous cell carcinomas were 0.88, 0.86, 0.87 and 0.84, respectively [8]. On the other hand, in another study aiming to classify temporomandibular joint osteoarthritis on cone beam computed tomography images using an artificial intelligence system, sensitivity, precision and F1 score values were found to be 1, 0.76 and 0.86, respectively. According to the results of our study, 1 image was estimated between 17.7ms and 19.2ms on a Tesla V100 16GB vram graphics card, and it does not seem possible for clinicians to detect a white spot lesion in 1 image in such a short time. In the YOLOv5x model used in this study, the ROC analysis gave a result of 0.712 AUC. The model’s performance metrics include a precision value of 0.786, a recall value of 0.618, and an F1 score of 0.692. However, most of the deep learning models used are semi-automatic, requiring manual data input and a certain amount of time, and suggest 1500 images and 10,000 examples (labeled objects) per class. In addition, the training here was done with a limited data set. The aim of this study is to evaluate the performance of the model after training with the limited number of data we have and to perform a preliminary study for future fully automatic models that can be clinically integrated.

In a pilot study by Askar et al. [6] to detect white spot lesions, lesions were classified as fluorotic and non fluorotic and automatically recorded with an accuracy of 81–84%. However, given the pixel imbalance between lesions, it was emphasized that hypoplasic changes such as the fluorotic group should be considered to have high degrees of granularity. Their labeling process theoretically allows for employing such Squeeze Net models, while given the expected pixel imbalance (only a minority of pixels are affected by the lesions) they assumed that for this type study, a classification model may be more feasible. Notably, they thought that it would also be relevant to assess the value of the ROI approach when performing segmentation modeling, as it may have more of a benefit for such task than in their study. It should investigate the accuracy of deep learning models for different severity levels of white spot lesions and extend multi-class detections towards more detailed disease classifications. The CNN developed by Schönewolf et al. [21] for automatic detection and classification of teeth affected by molar-incisor-hypomineralization (MIH) in intraoral photographs was able to accurately categorize teeth with MIH with an overall diagnostic accuracy of 95.2%. Based on these studies, it is technically feasible to develop CNNs with significant precision using software development. AI-based diagnostics will likely soon increase interest in dental photography in dentistry.

Dental photographs are used to determine treatment options between the patient and the physician, to select colors for aesthetic applications, and to transfer and archive images to the laboratory environment. While traditional usage of these photographs was primarily for patient records in orthodontics, their use in modern dentistry is expanding. In pediatric dentistry, digital photography employed mainly to find trauma, child abuse and neglect, caries, and dental abnormalities in children [22–26]. Orthodontic and pedodontic models photographed with appropriate shooting standards can be archived in the patient’s file and stored electronically for years [27].

Initial enamel lesions in the cervical regions can be seen in orthodontic patients after debonding, in patients with high caries risk, and in patients with early childhood caries [1, 28]. Even professional dentists may find it difficult to differentiate white spot lesions, making automatic recognition of these lesions in image databases or archiving systems crucial for diagnosis and treatment planning. In the principle of minimally invasive approaches, which have recently gained popularity, it is very important to diagnose and treat the first caries lesions at the microscopic level before cavitation occurs. In a study examining risk factors for early childhood caries in children with developmental enamel defects, it was found that the photographic method was more effective than clinical observation in identifying enamel defects [26]. Keeping photographic records and using an artificial intelligence system to diagnose and follow-up will help children with early childhood caries to continue to be examined and followed up by dentists even in the most familiar environments such as home environments, and to save significant time by not going to a clinic. Thus, dental anxiety and fears of children and their parents/caregivers can also be reduced [29]. Also, with using an artificial intelligence system such as Yolo algorithm, it is possible to evaluate the patient/parent education, preventive care monitoring, and post-treatment follow-ups of children undergoing orthodontic treatment and at risk of caries [30].

Despite the promising findings, this study has certain limitations. The intraoral photographs used were obtained after completion of oral examinations and prophylactic teeth cleaning. It is unclear whether the model can detect white spot lesions when plaque is present on tooth surfaces. Also white spot lesions can be mistaken for opaque enamel lesions caused by hypoplasia. To make a differential diagnosis, clinicians should thoroughly clean and dry the teeth before closely examining the lesions with a magnifying lens and sufficient lighting. While opaque enamel lesions caused by hypoplasia are typically smooth and shiny, white spot lesions are rough, opaque, and porous [1]. However, the appearance of the two lesions in photographs can be confusing due to lighting and reflections. To overcome this limitation, we have done to compare the lesion detected in the image to the patient’s initial treatment photographs and pay close attention to their localization to determine whether the lesion found in the picture is developmental or a white spot lesion. We here only present results from one specific model, YOLOv5x. Notably, model architectures emerge rapidly, and a more comprehensive benchmarking study could warrant more insight into which models to choose for different imagery types and modeling objectives.

Specifically, only white-appearing initial enamel lesions were labeled, while other initial enamel lesion marker tones were not included. Future studies may consider classifying all initial enamel lesion markers using a classification method such as the International Caries Diagnosis and Assessment System (ICDAS) [31] and integrating them into an artificial intelligence-based deep learning algorithm. This approach can provide valuable guidance for treatment options and improve the appearance of lesions after treatment. And also we anticipate that the limitations of this study can be overcome with the development of fully automated models with a high number of data and the use of different algorithms.

## Conclusion

The model’s accuracy and sensitivity in detecting white spot lesions remained lower than expected for practical application, but is a promising and acceptable detection rate compared to previous studies. The current study provides a preliminary insight to further improved by increasing the dataset for training, and applying modifications to the deep learning algorithm.

### Electronic supplementary material

Below is the link to the electronic supplementary material.

Supplementary Material 1

## Data Availability

The data is available from the corresponding author.
