# Supplementary figures and images for: Detecting white spot lesions on post-orthodontic oral photographs using deep learning based on the YOLOv5x algorithm: a pilot study
Source: BMC Oral Health. 2024 Apr 24;24:490. doi: 10.1186/s12903-024-04262-1 (PMC11044306; doi:10.1186/s12903-024-04262-1)

## 04/10/2023

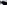

**This document has been signed with secure electronic signature.**

Supplement: Supplementary file 1 — Supplementary Material 1 [file 12903_2024_4262_MOESM1_ESM.pdf]
